# SDG 8 and the food–energy–water nexus: a two-country dynamic computable general equilibrium CGE model

**DOI:** 10.1186/s13705-022-00369-x

**Published:** 2022-10-23

**Authors:** Holger Schlör, Stefanie A. Schubert

**Affiliations:** 1 Forschungszentrum Jülich, 52425 Jülich, Germany; 2 SRH University Heidelberg, 69123 Heidelberg, Germany

**Keywords:** UN-SDG 8, General equilibrium model, Growth scenario, De-growth gap

## Abstract

**Background:**

In the twenty-first century, the success story of the Post-World-War-II World has been called into question by climate change and other challenges. De-growth or zero economic growth are discussed as possible solutions for mitigating climate change. The traditional economic growth model is increasingly challenged by the demand for sustained economic growth expressed in United Nations Sustainable Development Goal 8 “sustained economic growth” (UN-SDG 8) and supported by the European Green Deal. The aim of this paper is to contribute to the general understanding of characteristics, effects and challenges of new economic growth ideas as well as their interlinkages with the food–energy–water (FEW) nexus.

**Methods:**

To address these challenges, a stylized dynamic General Equilibrium Model (GEM) was developed, which consists of two countries: an emerging, developing European country A and a developed European country B. Country A is assumed to grow, while country B shrinks. The model is based on artificial data sets. This approach was chosen to prevent the blurring of counterfactual comparison by country-specific effects of economic turbulences such as the Lehman crash or the economic break-in during the Covid-19 pandemic.

**Results:**

The gross output of the emerging European country increases, whereas the output of the developed European country decreases according to the different growth strategies. The analysis reveals that a constantly widening gap between the emerging and the developed country is created. It can further be shown how this influences the relevant economic indicators (CO_2_ emissions, household budget, trade balance, utility and social welfare).

**Conclusions:**

The analysis of the two-country stylized GE model makes distortions visible: insignificant gaps in the values and development of analyzed economic indicators become prevalent. The welfare gap affects the core of the traditional socio-economic system, because the development of the utility of the households is central for the stability of political processes. A sufficiency and subsistence sector may be an option to even out the welfare losses from the de-growth strategy of the traditional economic system to avoid that the de-growth gaps are perceived by the community as welfare losses which can endanger the realization of UN-SDG 8.

## Background

The IMF Managing Director Kristalina Georgieva sees the success story of the Post-World-War-II [1] World threatened by climate change, inflation, inequality, the pandemic and the war in Ukraine [2, 3]. De-growth or zero economic growth as part of new economic growth models is being discussed as one option to solve the problems arising from climate change. The research question of this paper is to contribute to the understanding of characteristics, effects and challenges of new economic growth ideas as expressed also in UN Sustainable Development Goal 8 “Sustained, inclusive and sustainable economic growth” in detail as well as its connection to the FEW nexus. A subsection that completes this section shows that the FEW nexus plays a crucial role in the growth debate.

### Climate

Climate change is inextricably linked with the food–energy–water nexus, the key sectors of a sustainable development as described by the European Union [4]. Not only the European Union raises concerns about severe consequences of accelerating climate change [4], but also the German Climate Consortium summarized its latest findings on the threat of climate change in 2021: “Current policies would still result in a rise of around three degrees by the end of the century [5].” The global community is yet not on a zero-emission pathway. The German Cluster of Excellence Climate, Climatic Change, and Society (CLICCS) came to the conclusion that the necessary decarbonization of the global economy by 2050 is not only a technical problem, but above all a societal challenge [6]. The transformation process requires the definition of plausible political, economic, and cultural conditions under which the necessary transformations can be practically implemented [6].

### European Green Deal

The European Green Deal is the answer of the European Union (EU) to the challenges caused by accelerating climate change [7]. The aim of the EU is to be a fair and affluent society with zero GHG emissions in 2050, i.e., economic growth is decoupled from resource usage so that the decarbonization of the European countries can be fulfilled [8]. Therefore, the EU will renovate buildings, support the European industry to develop innovations and will change the private and public mobility. The new EU Green Deal of 2019 is combined with the Recovery Plan for Europe to enable a socio-economic recovery after the Corona pandemic [9–11]. The stimulus plan of the European Union includes financial expenses of over €2 trillion. With these two plans, the EU will modernize the European socio-economic system to enable a new economic growth strategy. However, in the European society, strategies that are more fundamental are being discussed to avoid severe climate change.

### De-growth and SDG 8

De-growth or zero economic growth is currently being discussed in science [12], politics [13] and documentary films [14–16] as one option to solve the issues addressed by Deaton, Stammer and the German Climate Consortium. As long as economic growth cannot be decoupled from resource usage to a sufficient extend, de-growth or zero-growth strategies have a key impact.

The discussion about alternative growth models is influenced and inspired by the work of Kenneth Boulding [17], who expressed ethical concerns about the future of the “cowboy economy” (i.e., an “economy of apparently illimitable resource”) already in 1966. He proposed instead the transition to a “spaceman economy” (i.e., an economy “without unlimited reservoirs of anything”) [17]. Boulding influenced also Nickolas Georgescu-Roegen “The entropy law and the economic process [18, 19] and Herman Daly “The economics of steady state” [20]. Based on these ideas, Tina Heikkinen discusses in her paper “A study of de-growth paths based on the von Neumann equilibrium model” based on the von Neumann model de-growth perspectives and the societal implementation of de-growth scenarios [21]. Mastini et al*.* developed their de-growth approach because of the restriction to keep global warming below 1.5 °C and achieve by 2050 a zero-emission society [22]. Ines Cosme et al. developed a framework to analysis the de-growth discourse [23]. Giorgos Kallis defends in his paper the de-growth approach because of the resource limits and the zero-CO_2_-emission restrictions to keep global warming below 1.5 °C [24]. Ulrich Brand describes the de-growth approach as a social movement which criticizes the current socio-economic system [25]. The international de-growth web portal sees the de-growth approach also as a critiques on the current global economic system [26].

In Germany, the idea of de-growth was pushed by the Study Commission of the German Parliament “Growth, Prosperity, Quality of Life—Paths to Sustainable Economic Activity and Social Progress in the Social Market Economy [13].” The report was written in the aftermath of the Lehman crises in 2008, the 2010 Euro crises and the initiated uncertainties about the future development of the Western economies and its challenges: the labor market, the financial markets, demographic change, rising public debts and the consequences of climate change, loss of biodiversity, the lack of intergenerational justice and social inequality [13]. These developments and challenges encourage the economic discussion about new prosperity ideas and welfare concepts. Steffen Lange summarizes in his book the macroeconomics of a world without economic growth. He gives a comprehensive overview of how de-growth economies can be sustainable [27].

Tim Jackson interprets the broad debate about post-growth scenarios as a “way of thinking about what might happen [28]” and as an invitation to analyze and explore new and historical social ideas for social progress [28]. In the view of Jackson, the post-growth debate is an opportunity to discuss socio-economic conditions beyond the current economic paradigm [28]. How the post-growth idea affects future economic development is the core of the research question.

The journey started on September, 25, 2015, as the General Assembly of the United Nations has adopted the 2030 Agenda for Sustainable Development made concrete through 17 Sustainable Development Goals (SDGs) [29]. The latter define 169 targets which aim to influence economic subjects towards more sustainable actions in various aspects over the next 15 years [29].

There are different perspectives on growth. On the one hand, the UN adopted SD Goal 8 as one of its Sustainable Development Goals (SDGs). SDG8 “promotes sustained, inclusive and sustainable economic growth, full and productive employment and decent work for all [29]”. In SDG 8, the UN expresses the need of sustained economic growth that is characterized by a high level of economic productivity and high resource efficiency in consumption and production to decouple economic growth from environmental degradation [29]. The UN growth concept includes decent work conditions and equal pay for women and men and an increase in youth employment. Thereby, the UN urges the end of forced labor and the protection through labor rights [29].

On the other hand, growth and sustainability are considered to be opposing goals. In this respect, the Post Growth Conference organizers and participants wrote an Open Letter to EU institutions to demand the end of the growth dependency of Europe [30]. The scientists wrote: “Growth at all costs divides society, creates economic instability, and undermines democracy [30].”

**Fig. 1:**
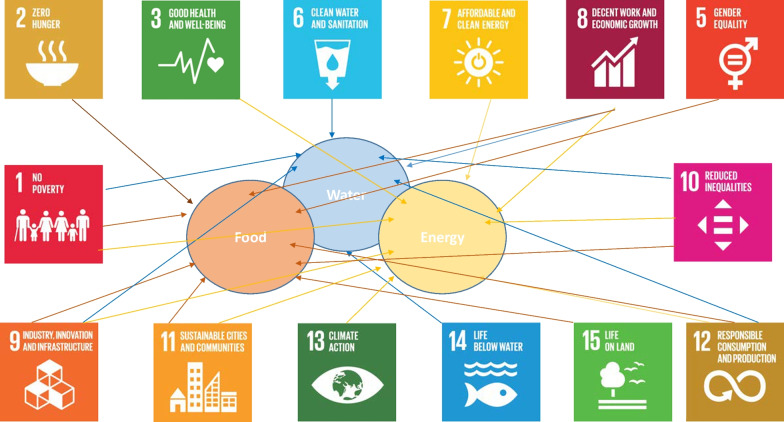
The network of the FEW nexus. Source: Authors, 2021 based on [29]

It is assumed that the different growth scenarios (unchanged economic growth, sustained economic growth (SDG 8) or de-growth scenarios) will have different impacts on all economic sectors, especially on the FEW nexus sector as the key sectors for a sustainable development, as the EU expressed it [4] and Fig. 1 demonstrates.

The FEW nexus sector comprises the key sectors of a sustainable development touching directly 14 of the 17 Sustainable Development Goals. The water sector is connected to seven SDGs (6, 8, 10, 12, 14, 9, 1), whereas the energy sector has connection to nine SDGs (3, 7, 8, 10, 12, 13, 11, 9, 1) and the food sector is also associated with nine SDGs (2, 8, 5, 10, 12, 15, 11, 9, 1).

All three FEW nexus issues are challenged by increasing demand and they are therefore under severe socio-economic pressure [4, 31]. This perspective was also emphasized by the Bonn 2011 Conference “The Water, Energy and Food Security Nexus” [32]. The Bonn conference supports the UN Conference on Sustainable Development "Rio 2012" and focuses on how the efficiency of the water, energy and food sectors can be increased, and the management of the sectors can be improved. The conference interpreted green growth as the sustainable usage of resources, providing clean water and energy and generate more rural jobs through green agriculture [32]. The conference established a connection between the nexus sectors and the idea of green growth.

The FAO supports the Bonn 2011 conference approach by regarding the water–energy–food nexus as a new management tool for the agricultural sector and of crucial importance for a decent life and a sustainable development [33]. The FAO cites Holger Hoff [34] by highlighting the link between the projected development of food, energy and water demand and population and economic growth [33]. The European Development Report takes up this linkage idea and emphasizes clearly the connection between the FEW nexus and the debate about new economic growth models [35].

### Growth debate and food–energy–water nexus

**Fig. 2:**
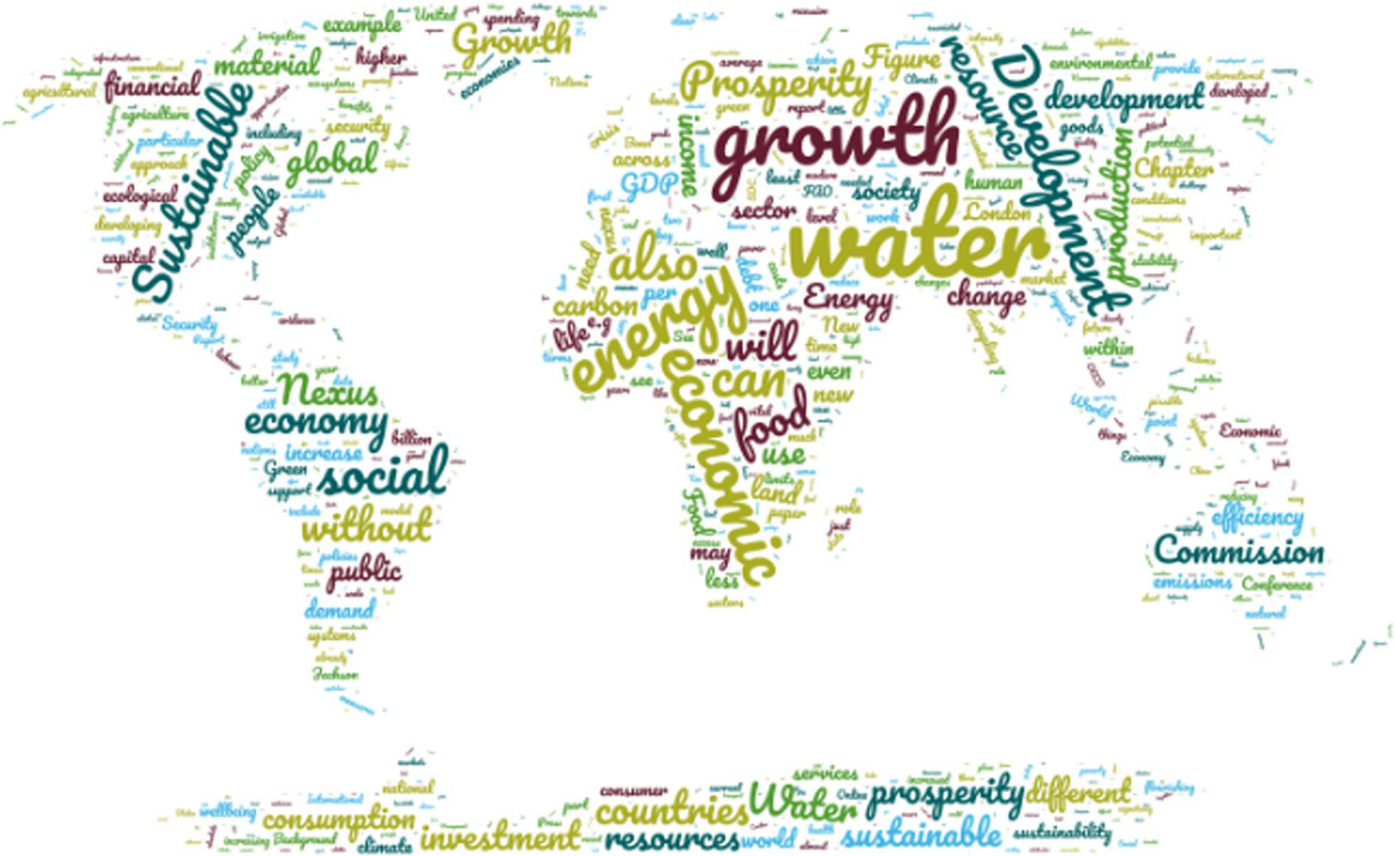
Economic growth and the FEW nexus. Source: Authors, 2021, own word cloud using https://www.wortwolken.com/

The scientific discussion about the design of a post-growth economy and the connection to the new food–energy–water nexus management concept is visualized in a text mining word cloud based on the FAO Report [33], the Bonn 2011 Conference Report [32], the Sustainable Development Commission [36] and the UN SDG Report [29]. The word cloud of Fig. 2 provides an overview of the current scientific discussion about the connection between the FEW nexus approach and the debate about new economic growth models.

The text mining of the four reports highlights the most frequent used keywords in the scientific discussion about the future economic growth concept and management of the food, energy, and water sectors. It reveals that water, energy and food are associated with prosperity and economic growth. The result reflects the current scientific and political discussion, which increasingly challenges the necessity and plausibility of the prevailing idea of economic growth [27].

Thus, research has to provide answers to the following research questions:

1. How is the FEW nexus as the key sector for sustainable development affected by different growth scenarios?
2. What are the economic implications arising from a system that is organized according to a zero or de-growth strategy?
3. How does this transformation process affect developing and developed European countries?

To address these challenges, a stylized, dynamic General Equilibrium Model is developed. It consists of two countries, a developing and emerging European country A and a developed European country B. The objective of the GE Model is to contribute to the understanding of characteristics, effects and challenges of new economic growth ideas in detail as well as its connection to the FEW nexus. The advantage and reason why this kind of analysis is applied is that it, first, covers direct effects that result from economic subjects’ optimization strategies in globalized economies with different growth rates. Second, a GE model also incorporates second-round indirect effects from economic subjects that are not affected directly but through decisions by other economic subjects.

This article proceeds as follows: In “Method—a two-country dynamic multinational CGE model” section, the model is presented. This includes the theoretical model description as well as the calibration of the model based on the database. In “Results” section, the results are presented in detail and in “Discussion” section, they are discussed. “Conclusions—design principles for the gap” section concludes with design principles for a new sector.

## Method—a two-country dynamic multinational CGE model

A multinational dynamic Computable General Equilibrium model (CGE model) is used [37–39] based on the general equilibrium economic theory of Walras (1834–1910) [38, 40] and the Ecomod model [37]. In its dynamic nature [41], it is based on the growth theory and models of Robert Solow [42] and Kaldor [43]. Solow explained that long-term economic growth can only be explained by technical progress [42, 44].

General equilibrium theory addresses the fact that an economy exists of many economic markets in complex interactions, where the action of every economic agent depends also on the acts of the other economic subjects [45]. For example, household demand for the various commodities of the economy depends on their income, which again depends on the wages, the profits of the company, which in turn depend on the technological level of the firm and its investment opportunities. The commodity prices are determined by demand and supply but depend on wages and firms’ profits as well [45].

Against this background, to conceptualize the new prosperity ideas of the UN-SDG 8 approach and the de-growth ideas, the presented CGE model based on the Ecomod basic model [37] covers a time frame of 15 periods (2020–2035) for two European countries (A, B). The design of the two countries is based on the systematic of the IMF.^1^ The countries represent a developing and emerging European country A and a developed European country B within the Euro area. The model is based on artificial data sets. This approach was chosen to prevent the blurring of counterfactual comparison by country-specific effects of economic turbulences such as the Lehman crash or the economic break-in during the Covid-19 pandemic. Trade relations [46] between the two countries are also analyzed as open economies are assumed. Additionally, it is assumed that all economic actors have rational forward-looking expectations [47], i.e., the behavior of the consumers and producers in both countries have expectations on the basis of the best information available at the time [48, 49].

A representative consumer in each country represents the consumption sector. This consumer maximizes its utility as in the neoclassical consumption model [37, 50]. To be precise, the consumer maximizes an intertemporal utility function based on the periodical utility *U*_*t*_, with *t* = 1,..,15.

1 $$U_{c} = \sum\limits_{t = 1}^{15} {\left( {\frac{1}{{1 + p_{c} }}} \right)^{t} \ln U_{t,c} } , \, \rho { = time \,preference \,rate, c = countries \,A + B , t = 1}..{15}$$

Periodical utility depends on the consumption C_t,c,i_ of goods from the different production sectors i over time t.

2 $$\begin{gathered} {\text{U}}_{t,c} = \prod\limits_{i = 1}^{3} {C_{ti}^{{\alpha_{c,i} Hi}} ,} \, \hfill \\ \alpha_{c,i} {\text{H}}_{i} \, {are \,the \,share \,parameters \,of \,both \,countries (c) } \hfill \\ {in \,the \,Cobb - Douglas \,utility}{. The \,sum \,of \,the \,share parameters \,equals \,1}{.} \hfill \\ \end{gathered}$$

The shares $\alpha_{c,i}$ and $1 - \alpha_{c,i}$ determine the consumption-per-period’s share in utility U_t,c_. Utility is maximized subject to the budget constraint:

3 $$\begin{gathered} Y_{t,c} = S_{t,c} + \sum\limits_{i = 1}^{I} {C_{t,i,c} } , \hfill \\ {{\text{t = period , c = country, i = sector,}}} \hfill \\ {\text{r}}_{{\text{t}}} \,{\text{is}}\,{\mkern 1mu} {\text{the}}\,{\mkern 1mu} {\text{interest}}{\mkern 1mu} \,{\text{rate}}{\mkern 1mu} \,{\text{of }}{\mkern 1mu} {\text{the}}\,{\mkern 1mu} {\text{two}}{\mkern 1mu} \,{\text{countries}}({\text{c}}) \hfill \\ \end{gathered}$$

In each period, the budget Y_t,c_ is spent on consumption and savings S_t,c_. From this optimization problem, the demand for goods can be calculated. Savings are assumed to finance investments.

The production sector of country c consists of three sectors. Each sector is represented by a firm, which operates under perfect competition and a constant returns to scale Cobb–Douglas [51] production function using capital K_t,c,i_ and labor L_t,c,i_:

4 $$XD_{c,t,i} = f_{c,t,i} \left( {K_{c,t,i}\,L_{c,t,i} } \right) = a_{c,i} F_{c,i} \cdot K_{c,t,i}^{{\alpha F_{c,i} }} \cdot L_{c,t,i}^{{\left( {\left( {1 - \alpha F_{c,i} } \right)} \right)}} , \, \alpha_{c,i} F + (1 - \alpha_{c,i} F) = 1.$$

The Cobb–Douglas function is homogeneous of degree one (linear homogeneous). This means that if labor and capital are increased by the factor t, then the output level would also increase by factor t. $a_{c,i} F_{c,i}$ represents the state of technology. The higher $a_{c,i} F_{c,i}$, the more efficient the employment of the production factors would be. Capital and labor are assumed internationally immobile. As $XD_{t,c,i}$ denotes the domestically produced output per sector and country in period t, this amount is supposed to meet domestic demand plus foreign demand, the exports. According to Armington [46, 52], it is assumed that imports and domestically produced goods are imperfect substitutes [53, 54].

The government is assumed to collect taxes and compensate households by granting various household transfers. Furthermore, the CGE model exhibits the following standard characteristics: demand of final goods and production factors is homogenous of degree zero in the price vector. Only relative prices matter according to Walras’ law. In other words, if n-1 markets are in equilibrium, the n^th^ market must be in equilibrium, too. Therefore, one of the market clearance conditions [55] are omitted.

### The social accounting matrix

The social accounting matrix (SAM) (Tables 1 and 2) represents the stylized status quo data set of each country of the model economy. The fictitious data sets of the two SAMs were compiled to illustrate and stress the effects of different growth scenarios in a model economy world unbiased by economic disruptions as the Lehman Brothers Collapse 2008, European debt crisis 2010 or the Corona pandemic.

**Table 1:** SAM emerging and developing European country A

Social accounting matrix country A—in currency units								
	FEW	Industry	Service	Consumption	Investment	Exports	Total	
FEW	0	0	0	170	12.5	40	222.5	
Industry	0	0	0	305	97.5	47.5	450	
Service	0	0	0	460	247.5	130	837.5	
Capital (K) payments	122.5	147.5	247.5					
Labor (L) payments	50	227.5	500					
Gross output (XD)	172.5	375	747.5					
Imports	52.5	75	90					217.5
Total	225.00	450	837.5					
						217.5		

Source: Ecomod, 2003 & authors, 2021

**Table 2:** SAM developed European country B

Social accounting matrix country B—in currency units								
	FEW	Industry	Service	Consumption	Investment	Exports	Total	
FEW	0	0	0	307.5	27.5	52.5	387.5	
Industry	0	0	0	545	205	75	825	
Service	0	0	0	1017.5	517.5	90	1625	
Capital (K) payments	247.5	325	497.5					
Labor (L) payments	100	452.5	997.5					
Gross output (XD)	347.5	777.5	1495					
Imports	40	47.5	130					217.5
Total	387.5	825	1625					
						217.5		

Source: Ecomod, 2003 & authors, 2021

It captures the full circular flow of transactions between the economic subjects [56, 57] and extends the traditional input–output approach. The data sets must be consistent and complete. Those requirements are fulfilled if income as represented by rows equal expenditures is represented by columns. Additionally, in the two-country setting, the imports of country A as shown in Table 1 equal exports of country B as shown by Table 2 and vice versa. It is assumed that both trade balances are balanced and there are no trade deficits or surpluses available. Each country has three economic sectors, a consumer and engages in international trade. The European emerging and developing country A is characterized by an agricultural, a service and an industrial sector. The developed European country B is characterized by a utility sector representing the energy and water sector, a service and industrial sector. The FEW nexus sectors are divided between the two countries.

Table 1 shows the stylized SAM of the European emerging and developing country A containing the expenditures for consumption, investment and exports and the capital and labor expenditures to enable the production of the gross output and the imports.

Table 2 presents the economic status quo of the developed European country B. The table shows that in all sectors, the total output of the developed European country B is higher than that of country A. Thus, the emerging country A is assumed smaller than the developed country B.

In both countries, the trade balance is balanced and exports equal imports.

### Calibration

The CGE model needs to be calibrated in order to reproduce the data set of the status quo as determined by the SAM correctly. This requires the determination of exogenous parameter values. Those include the steady state growth rate, the interest and time preference rate between the countries in order to elaborate specifically and exclusively the effects of the different growth models, as Table 3 shows).

**Table 3:** Exogenous parameters

Exogenous parameters		
Countries		
	Developing/emerging country A (%)	Developed country B (%)
Interest rate	5.0	4.0
Time preference rate	5.0	4.0
Steady state growth rate	2.5	− 1.0
Labor development	0.0	0.0

Source: Authors, 2021

IEK-STE/SRH 2021

- The emerging and developing country A will grow conventionally by 2.5% trying to follow the UN sustainable growth approach expressed in SDG 8. Country A grows in accordance with the growth rate of the emerging and developing countries in 2019, before the Corona pandemic [58].
- The developed European country B will shrink by 1.0%, based on the ideas and models developed by Jackson [59], Victor [12], Weitzman [60], Paech [61], Trainer [62] and D’Alisa [63, 64].
- It is assumed that the CO_2_-emission intensity (eta) of the two countries is different (Table 4). The CO2 intensity measures the CO2 emissions in relation to the production. The CO_2_ intensity of the developed country B is lower than that of the emerging country A, and the carbon intensity decreases by about − 1.8% for country A and about − 2.5% for country B. These assumptions are based on the World Development indicators of the World Bank [65]. Additionally, it is assumed that the CO_2_ intensity differs among the three sectors.

**Table 4:** CO2-emission intensity

CO2-emission intensity (eta) of the two countries and sectors			
kg CO2 per unit of gross output and consumption—in 2020			
Developing European country A			
	FEW	Industry	Service
Eta	0.40	0.41	0.42
Developed European country B			
	FEW	Industry	Service
Eta	0.18	0.19	0.2

Source: Authors, 2021

IEK-STE/SRH 2021

- A CO_2_ emission tax is assumed. The CO_2_ emission tax taxes the CO_2_ emissions related to gross output in addition to consumption CO_2_ emissions. The CO_2_ tax for **production** is 0.19 monetary units per kg CO_2_ for EU country A and 0.09 monetary units per kg CO_2_ in country B. The CO_2_ tax for **consumption** is 0.21 monetary units per kg CO_2_ for EU country A and 0.11 monetary units per kg CO_2_ in country B. The zero-growth initiative of country B is supported by lower CO_2_ taxes. The taxes are redistributed to the household of the respective country. The tax rate increases annually in the two countries with its specific economic growth rate: in country A by about 2.5% and in country B by 0.1%.

Each country has three production sectors: a service, an industry sector and a cross-country food–energy–water nexus sector. In the last sector, the agricultural sector is located in the emerging and developing country A, and the utility sectors (energy, water) are located in the developed European country B.

The service sector includes tangible and intangible services. The industrial sector consists of processing, manufacturing and construction companies. The companies sell machinery, equipment and supplies are used in manufacturing and construction.

Table 5 shows that the industry sector of the developed European country B has the highest technological level, followed by the service and utility and agricultural sector of country B. The initial efficiency level of the developed country B is higher than in country A in every sector. The efficiency parameter is the lowest for the agricultural sector of the emerging and developing country A.

**Table 5:** Technological efficiency parameter

Technological efficiency parameter, aF			
Developing/emerging country A		Developed country B	
Agriculture	0.424	Utilities	0.702
Service	0.870	Service	1.127
Industry	0.955	Industry	1.210

Source: Own calculations, 2021

IEK-STE/SRH 2021

## Results

Here, the results of the stylized model for the emerging and developing country A and developed European country B will be presented. The impact of the proposed growth paths in countries A + B will be broken down to further analyze all relevant economic variables. This includes the key economic indicators such as gross output, utility level and social welfare, and CO_2_ emissions, which are an indicator for the decarbonizations of the two-country economy. Decarbonization is a central element of the EU Green Deal [7, 8].

### Gross output

**Fig. 3:**
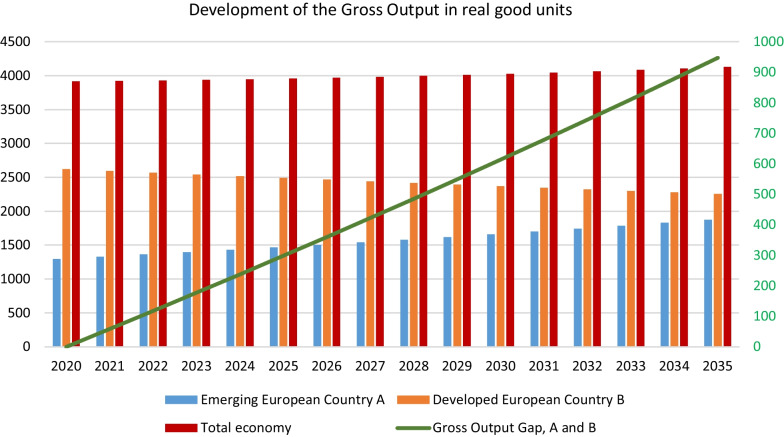
Gross output. The gross output of the emerging European country increased from 1295 units in 2020 to 1876 units in 2035, whereas the output of the developed European country decreased from 2620 units in 2020 to 2253 units in 2035, so that the total output of the two-country economy increased from 3915 to 4129 units in 2035. The decrease of the gross output of the developed country is more than even out by the gross output of the emerging country. The total gross output increased and created a growing output gap between the two countries. The gap resulting from the output declines in the three sectors depending on their initial size. Figure 4 shows the absolute size of the total and sectoral gaps

**Fig. 4:**
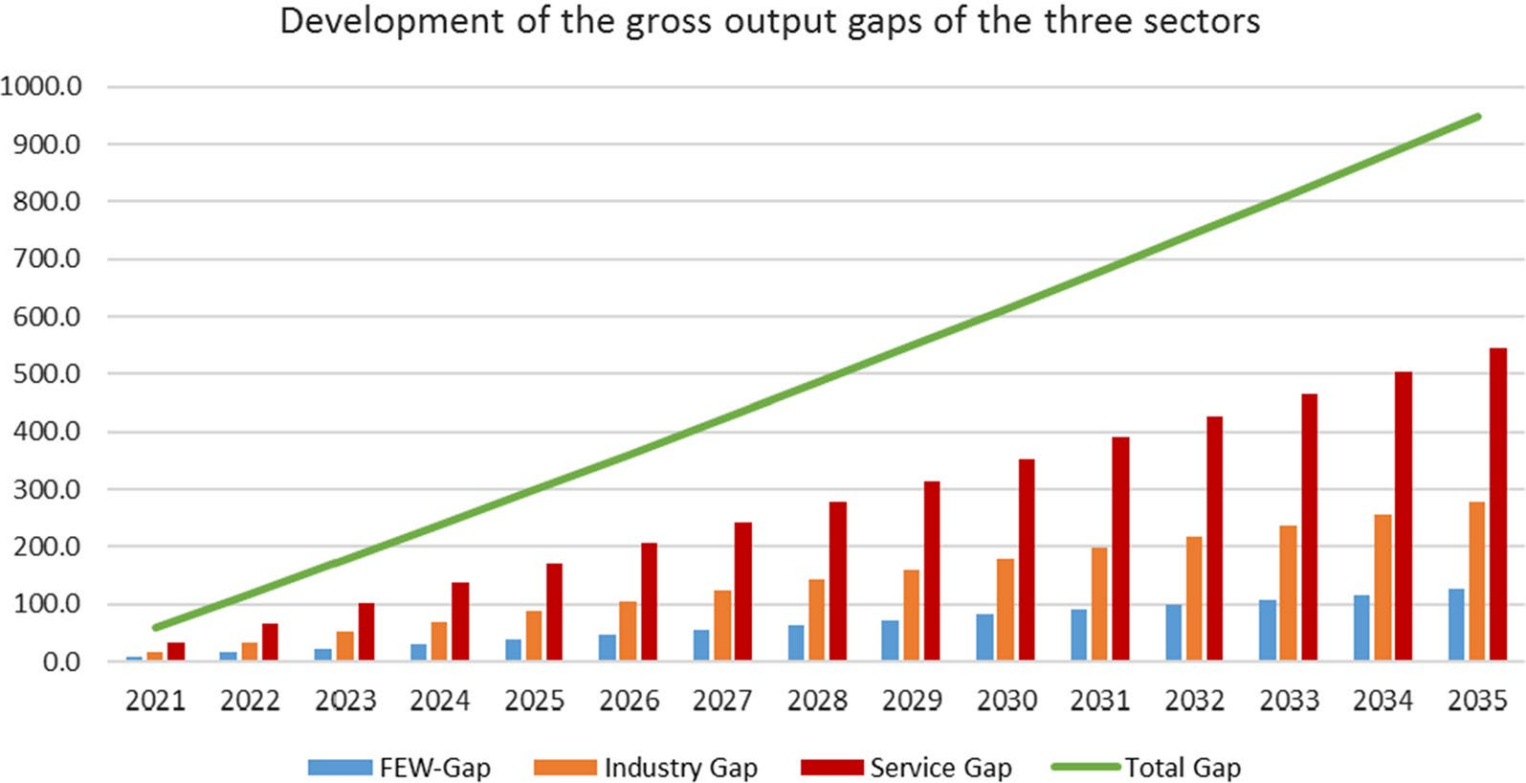
The highest increase in the gap takes place in the service sector (+ 544 units) followed by the industry (+ 277 units) and the FEW nexus sector (+ 126 units). This development created an increasing output gap between the two countries of about 947 units

Figures 3 and 4 depicts the gross output—the measure for the economic performance of the two countries in the production of goods and services—of the two different economies and the respective sector outputs. The analysis reveals that different growth strategies imply diverging directions of the two countries creating a gap between the emerging and the developed country. It also shows how the different growth strategies unfold differences at the sector level.

### Trade balance

The developments presented so far also have an impact on the trade relationship of the two countries: the trade balance. The trade balance is the difference between national exports and imports in monetary values over a certain period.

The exports of the emerging country A increase from 218 monetary units in 2020 to 315 in 2035, whereas the exports of the developed country B decrease in the observed period from 218 to 187. The imports of both countries develop analogously, so that both countries’ trade balances are balanced.

**Fig. 5:**
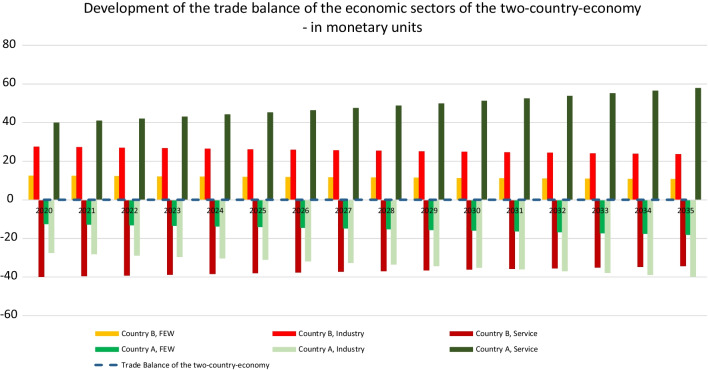
In the following, the development of the CO_2_ tax is analyzed

The following, Fig. 5 takes a deeper look at sectoral results. The trade surplus of the FEW sector of country B decreased from 12.5 to 10.8 monetary units in the observed period, whereas the trade surplus of the industry sector of country B decreased by about 3.8 units. The service sector of country B can reduce its trade deficit by about 5.6 units. The FEW sector of country A increases its trade deficit by about 5.6 units and its industry sector increases its deficit by about more than 12 units, whereas the service sector of country A expands its trade surplus to about nearly 18 units, so that the following picture of the trade relations of the two countries over the observed time period is built.

### CO_2_ tax

The development of the CO_2_ tax is based on the development of the greenhouse gas CO_2_ emissions over the observed 15-year period. CO_2_ emissions are calculated based on the CO_2_-emission intensity of the two countries: CO_2_ emissions per unit gross output. The emission intensity of the two countries differs due to the different technological level, as defined in chapter 2. The CO_2_-emission intensity factor of the developed country is taken from the 2019 CO_2_ emissions of the EU-28 intensity factor (0.172). The CO_2_-emission intensity factor of the emerging country A equals the value determined by the IEA in 2019 for OECD countries (0.23).^2^

The total CO_2_ emissions of both countries grew by about 8.7%, whereas the total emissions of emerging country A increased by about 40% and those of the developed country B decreased by about -13.4%. The emissions of the three sectors of the emerging economy of country A increased by about 45.4% in the service sector and by about 43% in the agricultural sector and by 39.6% in industry. The emissions of the developed country B declined between 12 and 13% in the 3 sectors.

**Fig. 6:**
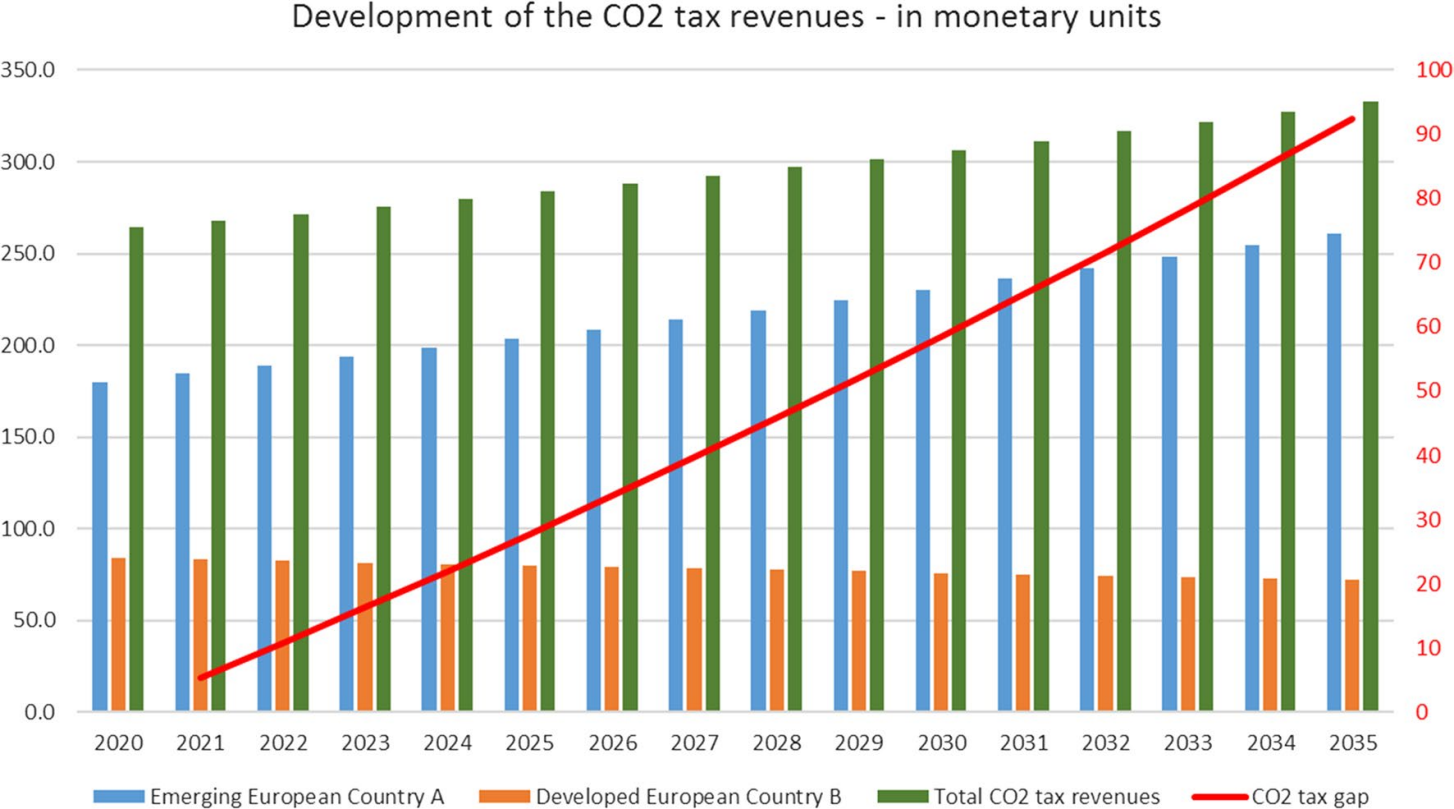
CO2 tax revenues. The total CO2 tax revenues of the two-country economy increased from 264.1 to 333 units and the CO2 revenue gap between the two countries increased to 92.45 monetary units (Fig. 6). The significant difference of the CO2 tax revenues of the two countries are caused by the different technological levels expressed in Table 5, the different respective CO2intensities (Table 4), and the different growth rates of the CO2 tax in the analysed period. The CO2 tax in country B is lower than in country A as an institutional appreciation for its strategy of declining economic growth.

**Fig. 7:**
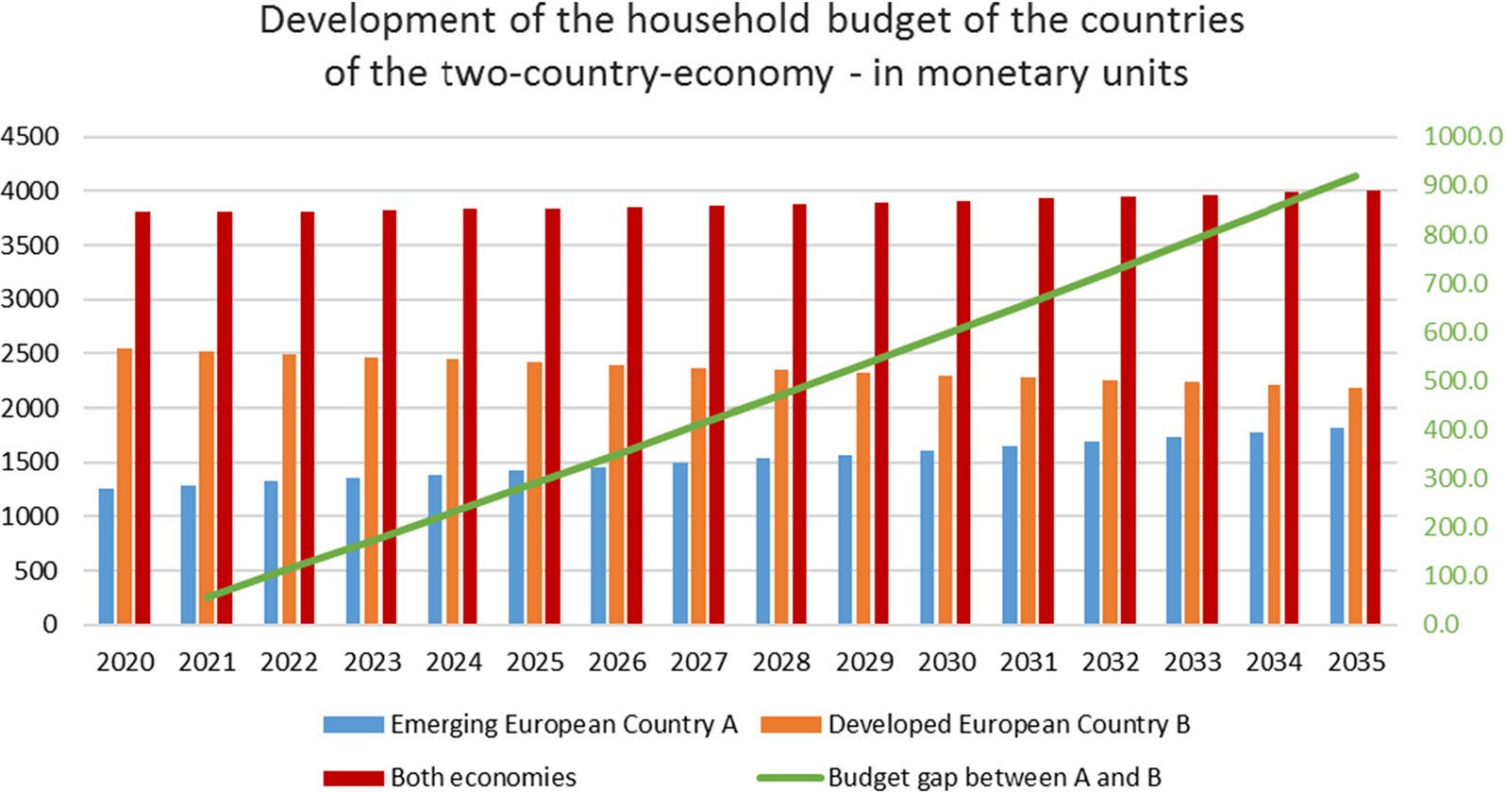
Utility and social welfare. The gap between the two countries increased to about 920 monetary units (Fig. 7), so that at the end of the analysis period the household budget increased by about 14% for the households of country A and about 3% for the households of country B.

**Fig. 8:**
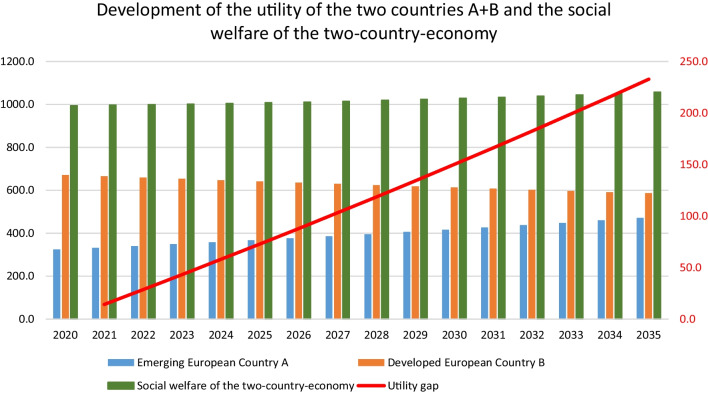
Utility and social welfare. The utility level of the emerging country A increases by about nearly 40%, whereas the utility level of the developed country B declines slightly by about 13% over the 15 years analyzed so that the social welfare of the two-country economy increased by about 6.3% over the observed period. The decline of the CO_2_ emissions of country B is accompanied by a decline of the utility of the country. The elasticity of substitution expressed as the utility loss caused by the taxation of CO_2_ increased from 0.7047 in 2020 to 0.7096 in 2021 (Fig. 9)

**Fig. 9:**
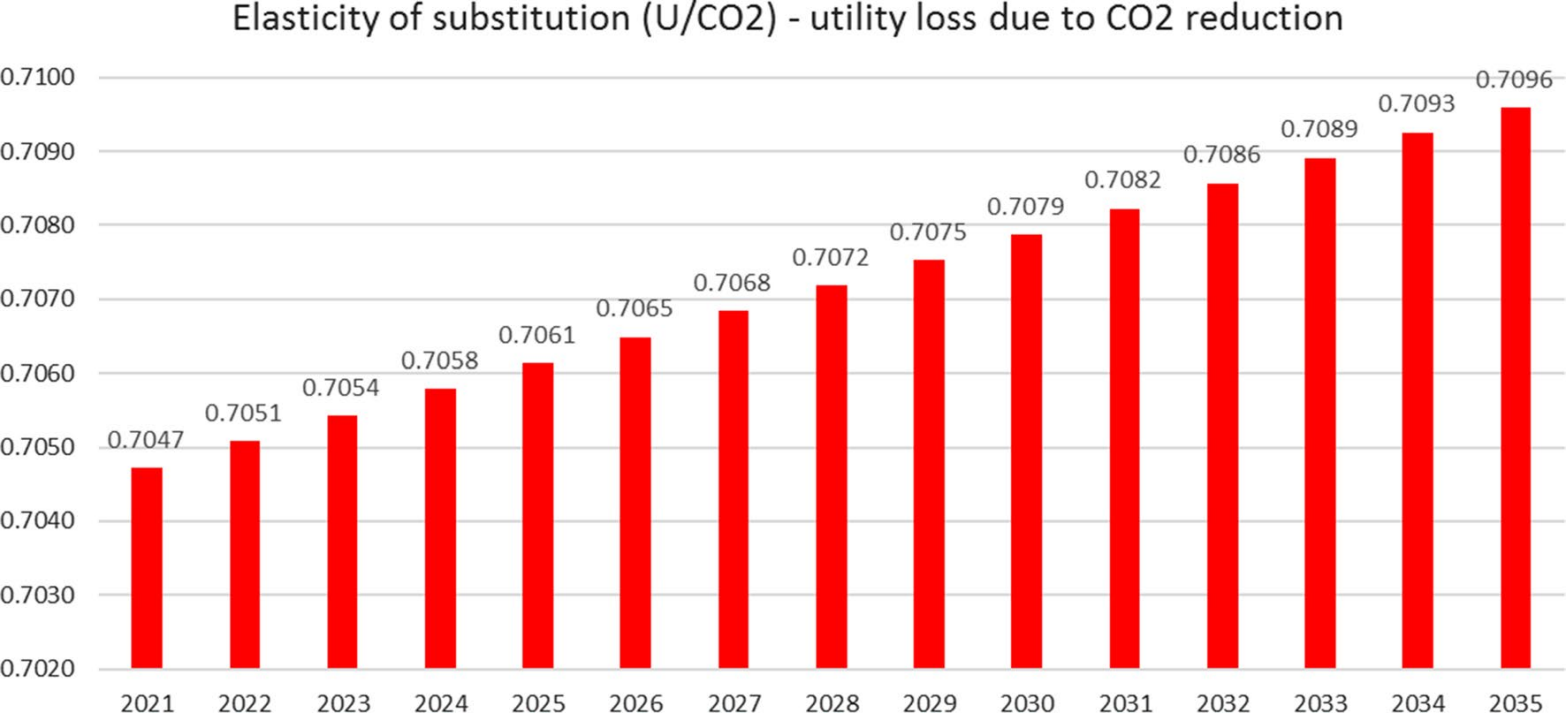
Elasticity of substitution. The reduction of one unit of CO_2_ causes a reduction of the utility level of country B by about 0.7096 in 2035. The elasticity of substitution increased annually by about 0.05% and over the analyzed time by about 0.69%. A growing utility gap can influence the political systems, if the consumers of country B perceive the utility decline as a welfare loss. First, it is well known that people compare themselves to others of the peer group [67]. Thus, the consumer of the developed country B might perceive the gap of international development negatively. Second, the perception might be even more negative, as the loss, which consumer of Country B faces, is more important than a gain with changes always being related to reference points according to the prospect theory by Kahneman and Tversky [68]

CO_2_ tax revenues of country A increased from 180 monetary units in 2020 to 260.6 unity in 2035, whereas the tax revenues of country B decreased by about 11.7 units to 72.3 units in the observed period (Figs. 6, 7, 8 and 9).

### Household budget

Each household budget—the amount of money that is available for the households to spend for consumption—includes earned income and CO_2_ tax compensation payments. The household budget of the emerging European country A increased over the 15-year period by about 44.83%, whereas the income of the developed country B decreased by about 14%, building an income gap between the two countries, whereby the total generated income of both countries increased by about 5.47%.

## Discussion

The development of the discussed key economic indicators has an influence on the stability of the political system of the two countries. How the citizens of the two countries perceive—measured by the utility indicator—the development of the key indicators based on the different growth models will determine whether they support the political measures of de-growth or zero-growth economic development.

Therefore, in the following the impact of the development of the key economic indicators on the welfare of the households of the two-country economy is discussed. Thereby, the question will be discussed whether a social optimum between reduction of negative ecological effects and minimization of possible welfare losses can be achieved by new growth models?

### Utility and social welfare of the two-country economy

The development of the social welfare (SWF) of the two countries can be derived from the utility of the representative consumer according to Eq. 1. The economic parameter utility refers to the degree of satisfaction of needs that households derive from consumption of goods and services based on the theories of Jeremy Bentham and Jean Walras [66]. The social welfare is the sum of utility of the two-country economy:

5 $$\begin{gathered} {\text{SWF = }}\sum\limits_{{j = 1}}^{2} {{\text{U}}_{{t,c,j}} } = \prod\limits_{{i = 1}}^{3} {C_{{ti}}^{{\alpha _{{c,i}} Hi}} ,} {\text{ j = countries A and B}} \hfill \\ \alpha _{{c,i}} {\text{H}}_{i} {\text{are}}\;{\mkern 1mu} {\text{the}}{\mkern 1mu} \;{\text{share}}{\mkern 1mu} \;{\text{parameters}}\;{\mkern 1mu} {\text{of}}\;{\mkern 1mu} {\text{both}}{\mkern 1mu} \;{\text{countries}}{\mkern 1mu} {\text{(c) in}}\;\hfill \\ {\mkern 1mu} {\text{the}}\;{\mkern 1mu} {\text{Cobb - Douglas}}{\mkern 1mu} \;{\text{utility}}.\,{\text{The}}\;{\mkern 1mu} {\text{sum}}{\mkern 1mu} \;{\text{of}}{\mkern 1mu} \;{\text{the}}\;{\mkern 1mu} {\text{share}}\;{\mkern 1mu} {\text{parameters}}\;{\mkern 1mu} {\text{equals}}\;{\mkern 1mu} {\text{1 }} \hfill \\ \end{gathered}$$

The economic development of the two countries causes also a drifting of the utility level of the consumers of the two countries (Fig. 8).

### Development gaps between emerging country A and developed country B

**Fig. 10:**
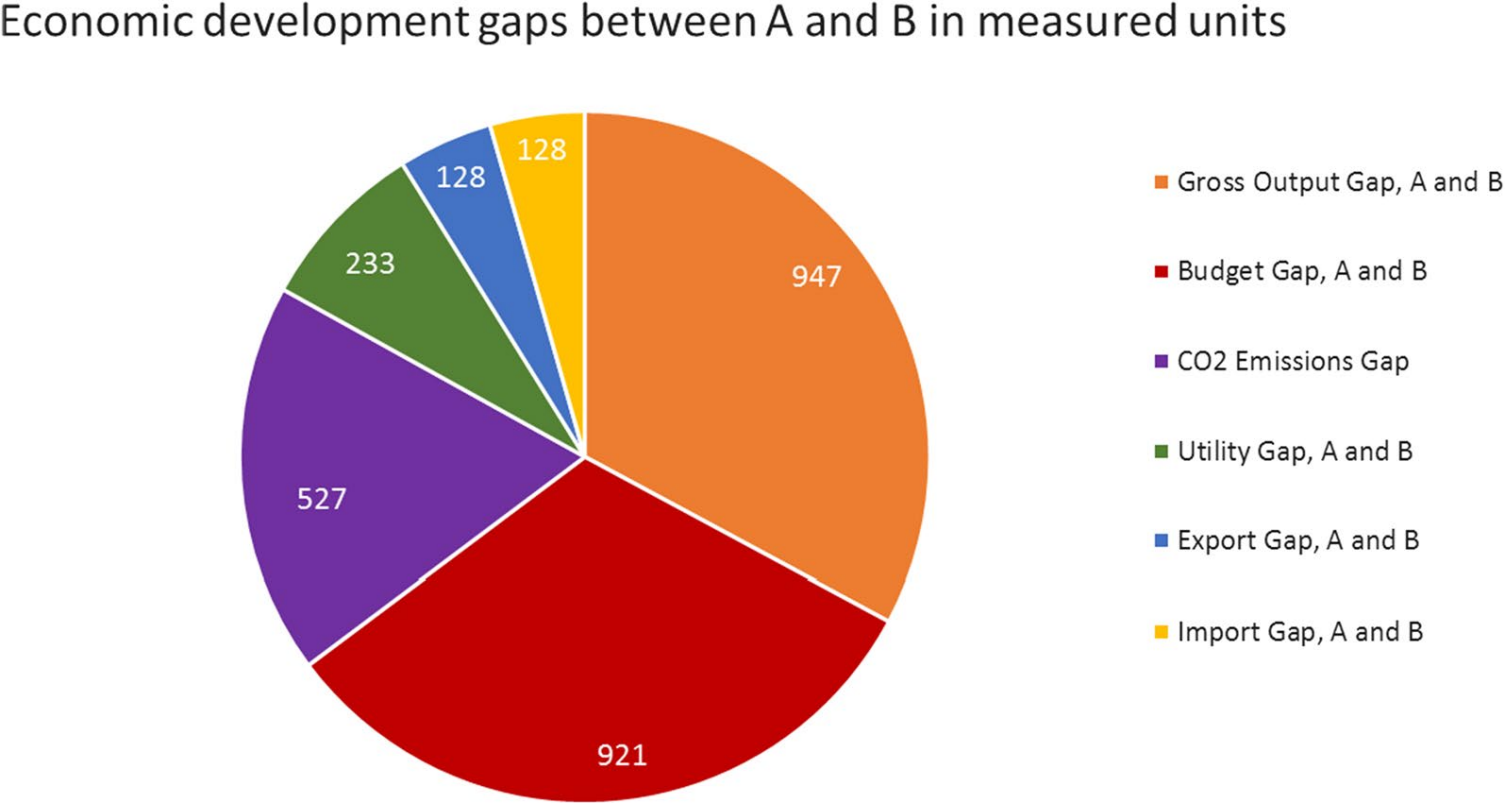
Economic gaps. The greatest gap over the observed period (2020–2035) is caused by the development of the gross output (947 real units) in the development of the income (921 monetary units) of the two countries. The CO_2_ emission gap (527 units) is a little bit lower than that of the consumption, followed by the utility gap (233) and the trade gap between the countries (128)

The negative-growth strategy of the developed country B causes development gaps between the two countries of the two-country economy (Fig. 10).

**Fig. 11:**
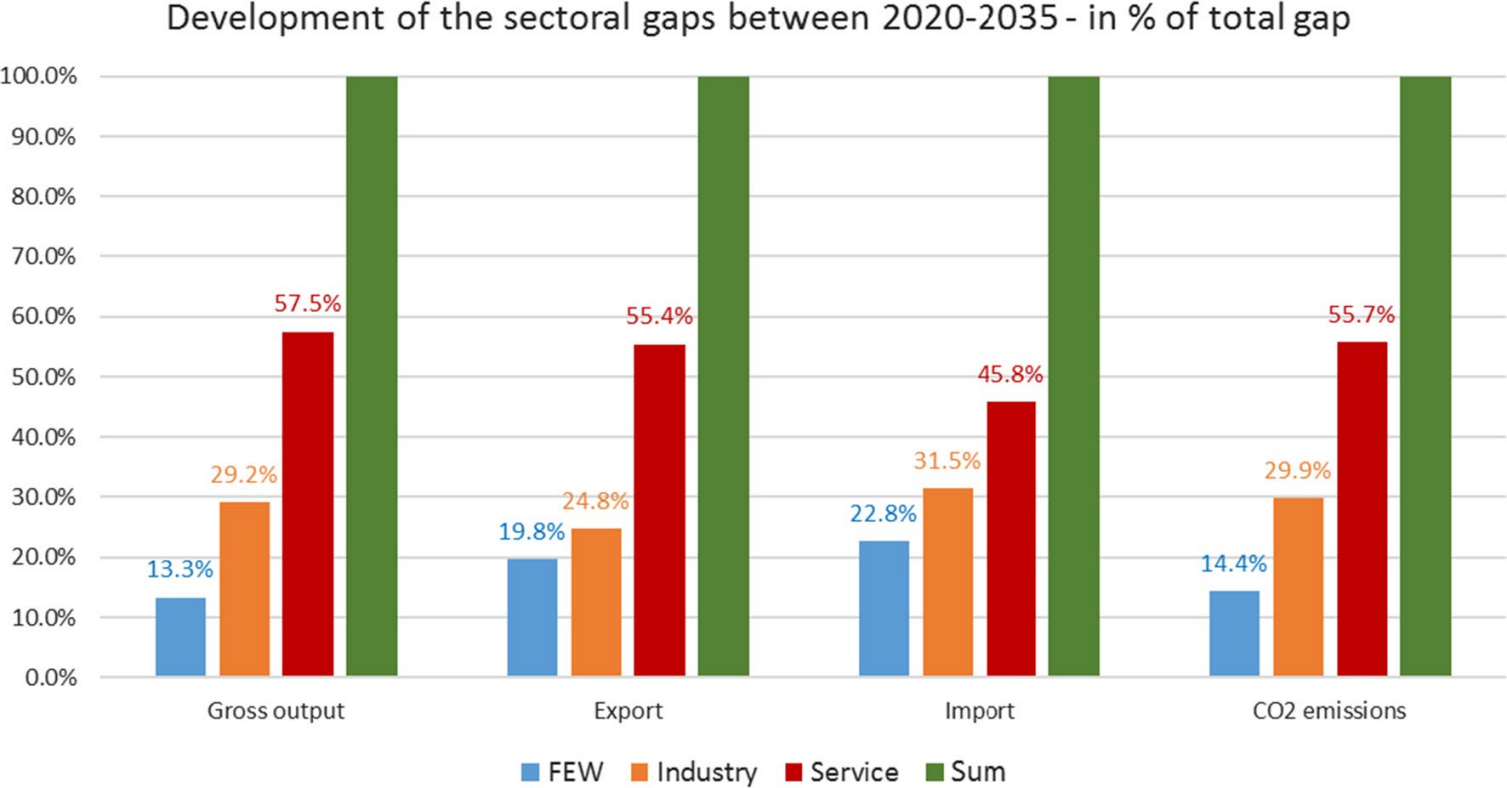
Sectoral gaps. The gross output gap is caused by more than 50% by the service sector (57%) followed by the industrial sector (29%) and the FEW nexus sector (13%). The export and import gaps differ significantly in the service sector by about nearly 10 percentage points (55.4% and 45.8%); this difference is reduced in the case when the industry sector reaches seven percentage points. The FEW sector showed the lowest difference with 3 percentage points. CO_2_ emissions demonstrated a similar distribution of the sectoral gaps as the gross output

Figure 11 shows which sectors are responsible for the development of the gaps between the two countries.

**Fig. 12:**
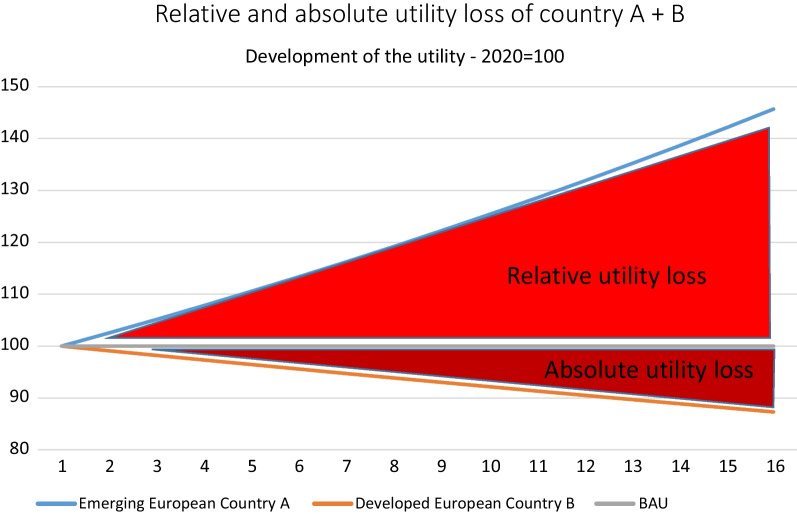
Utility level country A and B. It can be summarized for all analyzed economic indicators that the FEW nexus sectors are causing the lowest gap of all three sectors, followed by the industry and the service sector

The analysis of the two-country stylized GE model reveals distortions arising from differing growth, which are made visible by the gaps of the analyzed economic indicators. The size of the gaps differs for the various economic indicators. The utility and welfare gap affected the core of the traditional socio-economic system, as for the implementation of a de-growth society, the development of the utility of the households is central for the political process and the necessary societal support. Figure 12 indicates that the two-country economic system achieved a utility plus of 6.3% in the 15-year period, whereas country A achieve a utility plus and country B has to face a relative and absolute utility loss. The utility of country B declines in comparison to period 1 by about 12.7% and declines relatively by to about 59% with regard to the development of country A. Country B’s utility contracted in absolute and relative terms, which can be seen below.

## Conclusions—design principles for the gap

The presented model results are causing the following questions:

- How would the households of country B assess the relative and absolute losses of utility?
- Does society perceive the decline of benefit as a loss that has to be balanced by other economic activities as suggested by Tim Jackson and N. Paech?
- Which societal institutions can organize or initiate the measures to even out the utility loses?
- Would society accept the utility decline as a price for the net-zero-emission society?

The result is the growth sustainability paradox: if economic growth is reduced, the CO_2_ emissions and the household utility can decline and can thereby endanger the stability of democracy. A sufficiency and subsistence sector may be an option to even out the welfare losses from the de-growth strategy of the traditional economic system to avoid that the de-growth gaps are perceived by the community as welfare losses.

This sufficiency and subsistence sector can be interpreted as a common good sector and a first institutional step towards a societal optimum between the reduction of negative ecological effects and the reduction of welfare losses. This sector also needs principles, rules and institutions to even out the welfare losses caused by the negative-growth strategy. It is important that a country develop measures to even out the welfare losses of the consumers to avoid that the reduced utility level will influence the political system of the country. Country B has to develop a sustainable socio-economic-ecological system in the SDG framework to avoid a growing utility gap causing distribution struggles in society.

The presented model has the advantage that more countries and more problems expressed by the IMF Managing Director can be included in the model. This would require developing a consistent data set including the social accounting matrix, country-specific technological efficiency parameter, CO_2_-emission intensity and the specific trade relations.

## Data Availability

Not applicable, there is no further data used in this study.

## Footnotes

1 https://www.imf.org/external/datamapper/NGDP_RPCH@WEO/OEMDC/ADVEC/WEOWORLD.

2 https://www.deutschlandinzahlen.de/tab/welt/umwelt-energie/umwelt/co2-emissionen-in-kg-pro-bip-einheit.
